# Inclusion of postdoctoral trainees in a translational science training TL1 program was associated with greater diversification of research across the translational science continuum: a bibliometric analysis of TL1 trainee publications

**DOI:** 10.1017/cts.2023.603

**Published:** 2023-07-28

**Authors:** Amanda M. Moore, Linda M. McManus, Yong-Hee P. Chun, Julie Barker, Laura D. Davis, Jillian Rosenzweig, Esther Albuquerque, Zaynab Omisade, Bruno Onwukwe, Christopher R. Frei

**Affiliations:** 1 College of Pharmacy, The University of Texas at Austin, San Antonio, TX, USA; 2 Long School of Medicine, University of Texas Health San Antonio, San Antonio, TX, USA; 3 School of Dentistry, University of Texas Health San Antonio, San Antonio, TX, USA; 4 South Texas Veterans Health Care System, San Antonio, TX, USA

**Keywords:** translational science, tl1, T32, nih training program

## Abstract

The NIH National Center for Advancing Translational Science (NCATS) was established to support translational research that spans the entire TS Continuum, with the goal of bridging the gap between preclinical biomedical research and real-world applications to advance treatments to patients more quickly. In 2018, the Translational Science Training (TST) TL1 Program at the University of Texas Health Science Center at San Antonio implemented new strategies to better include and encourage research more broadly across the TS Continuum, including the addition of postdoctoral scientists and a clinically trained Program Co-Director, expansion of team science and community engagement programming, and targeted trainee recruitment from schools of nursing, dentistry, and allied health, in addition to medicine. The objective of this bibliometric analysis was to determine if the program exhibited a more diverse mix of T-types after the adjustments made in 2018. The TST/TL1 Program experienced a shift in T-type, from mostly T0 (preclinical) to more T3/T4 (clinical implementation/public health) research, after new strategies were implemented. This supports the conclusion that strategic programmatic adjustments by an NCATS-funded predoctoral training program resulted in outcomes that better align with NCATS priorities to develop Trainees who contribute across the entire TS Continuum.

NIH’s National Center for Advancing Translational Science (NCATS) aims “to turn biomedical research discoveries into health solutions…through the application of translational science (TS) [1].” NCATS recently developed TS Principles [1,2] based on information from its Clinical and Translational Science Award (CTSA) Education Core Competency Working Group and a publication regarding “The Fundamental Characteristics of a Translational Scientist [3],” to guide its education and training initiatives and to describe the knowledge, skills, and abilities necessary to facilitate translation across the TS Continuum [1].

The Translational Science Training (TST) TL1 Program at the University of Texas Health San Antonio was established in 2009 in affiliation with the Institute for the Integration of Medicine and Science – home for the institution’s CTSA – and offers TL1 support for multidisciplinary research and education for predoctoral and postdoctoral scientists.

In 2018, the TST/TL1 Program implemented several new strategies to better include and encourage research more broadly across the TS Continuum, including the addition of postdoctoral scientists and a clinically trained Program Co-Director, expansion of team science and community engagement programing, and targeted trainee recruitment from schools of nursing, dentistry, and allied health, in addition to medicine. The objective of this bibliometric analysis was to categorize publications of TST/TL1 trainees according to Surkis’ TS “T-types” (T0–T4) [4] and to determine if the TST/TL1 Program exhibited a more diverse mix of T-types after the programmatic adjustments made in 2018.

Bibliometric information was gathered for all TST/TL1 trainees who participated in the program from 7/1/2009 to 12/31/2021 using PubMed, Scopus, and Google Scholar searches. Original research articles qualified for inclusion in this analysis when a TST/TL1 trainee was listed as a coauthor in any position and when the work was published after they were appointed to the TL1 training grant. Editorials, commentaries, and errata were excluded. One trained investigator independently reviewed the title and abstract for each published article and classified the article based on the T-type criteria outlined by Surkis *et al* [4]. T-types were categorized as T0 (preclinical), T1/T2 (clinical), and T3/T4 (implementation/public health). A second trained investigator independently repeated the process for each article. When the first two independent ratings differed, a third trained investigator independently repeated the process. Once two independent ratings were the same, the classification process was complete. Percentages of publications belonging to the different T-types were plotted by publication year to assess if diversification of research occurred as time progressed in the TST/TL1 Program. Percentages were plotted by publication year, rather than TL1 cohort year, because trainees were appointed to the TL1 training grant for different lengths of time (including one trainee who was appointed in both the pre- and post-periods). Also, many TST/TL1 Program activities were open to all predoctoral and postdoctoral scientists, and it was common for them to participate in TST/TL1 Program activities before and after they were officially appointed to the TL1 training grant. The TST/TL1 Program design at the University of Texas Health Science Center at San Antonio might differ from TL1 program designs at other institutions regarding how many program activities are open to all, even if they are not officially appointed to the TL1 grant.

Publications were subsequently divided into pre- (2009–2017) and post- (2018–2021) periods to assess differences in T-types before and after the program modifications. Groups were compared using chi-square and Fisher’s exact statistical tests.

Overall, 494 publications from 77 TST/TL1 trainees were included in this analysis. In 2010, 100% of publications by trainees were T0; however, by 2021, over half of the publications by trainees were other T-types (Fig. 1): T0 (48%), T1/T2 (13%), and T3/T4 (39%). When comparing T-types for the publications in the pre- (2009–2017) and post- (2018–2021) periods, the percentage of T0 publications was significantly lower in the post-period (89% vs 66%, *p* < 0.01), the percentage of T1/T2 was similar in both periods (6% vs 8%, *p* = 0.48), and the percentage of T3/T4 publications was higher in the post-period (5% vs 27%, *p* < 0.01).

**Figure 1.**
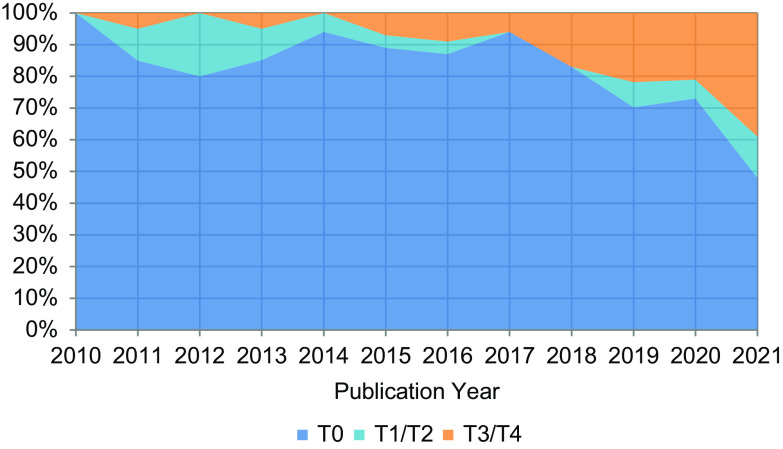
Annual distribution of TST/TL1 trainee publications by T-type.

The Translational Science Training (TST)/TL1 Program experienced a shift in T-type, from mostly T0 (preclinical) to more T3/T4 (clinical implementation/public health) research, after new strategies were implemented. A 2019 survey of TL1 programs by Sancheznieto *et al* [5] reported that predoctoral programs were more likely to have trainees engaged in basic (51% vs 43%) and T0 (preclinical) (76% vs 65%) research, but less likely to have trainees engaged in T1/T2 (clinical) (76% vs 89%), T3 (implementation) (61% vs 68%), and T4 (public health) (61% vs 65%) research, as compared to postdoctoral programs. This suggests that the inclusion of postdoctoral trainees in the TST/TL1 Program (0% in the pre-period and 31% in the post-period) contributed to the greater diversity of T-types in the post-period. These two studies underscore the need for NCATS to continue supporting TL1/T32 postdoctoral training programs if these programs are to produce trainees who work along the TS Continuum.

The findings of this analysis suggest that strategic programmatic adjustments, such as the inclusion of postdoctoral trainees, were associated with a more diverse mix of TS activity in the post-period, thereby helping the TST/TL1 Program at the University of Texas Health Science Center at San Antonio to better reflect research along the TS Continuum.
